# Exploring the context in which different close-to-community sexual and reproductive health service providers operate in Bangladesh: a qualitative study

**DOI:** 10.1186/s12960-015-0045-z

**Published:** 2015-09-01

**Authors:** Ilias Mahmud, Sadia Chowdhury, Bulbul Ashraf Siddiqi, Sally Theobald, Hermen Ormel, Salauddin Biswas, Yamin Tauseef Jahangir, Malabika Sarker, Sabina Faiz Rashid

**Affiliations:** James P Grant School of Public Health, BRAC University, Dhaka, Bangladesh; Department of International Public Health, Liverpool School of Tropical Medicine, Liverpool, UK; Royal Tropical Institute, Amsterdam, The Netherlands

**Keywords:** Close-to-community health service providers, Informal health service providers, Sexual and reproductive health, Menstrual regulation, Bangladesh

## Abstract

**Background:**

A range of formal and informal close-to-community (CTC) health service providers operate in an increasingly urbanized Bangladesh. Informal CTC health service providers play a key role in Bangladesh’s pluralistic health system, yet the reasons for their popularity and their interactions with formal providers and the community are poorly understood. This paper aims to understand the factors shaping poor urban and rural women’s choice of service provider for their sexual and reproductive health (SRH)-related problems and the interrelationships between these providers and communities. Building this evidence base is important, as the number and range of CTC providers continue to expand in both urban slums and rural communities in Bangladesh. This has implications for policy and future programme interventions addressing the poor women’s SRH needs.

**Methods:**

Data was generated through 24 in-depth interviews with menstrual regulation clients, 12 focus group discussions with married men and women in communities and 24 semi-structured interviews with formal and informal CTC SRH service providers. Data was collected between July and September 2013 from three urban slums and one rural site in Dhaka and Sylhet, Bangladesh. Atlas.ti software was used to manage data analysis and coding, and a thematic analysis was undertaken.

**Results:**

Poor women living in urban slums and rural areas visit a diverse range of CTC providers for SRH-related problems. Key factors influencing their choice of provider include the following: availability, accessibility, expenses and perceived quality of care, the latter being shaped by notions of trust, respect and familiarity. Informal providers are usually the first point of contact even for those clients who subsequently access SRH services from formal providers. Despite existing informal interactions between both types of providers and a shared understanding that this can be beneficial for clients, there is no effective link or partnership between these providers for referral, coordination and communication regarding SRH services.

**Conclusion:**

Training informal CTC providers and developing strategies to enable better links and coordination between this community-embedded cadre and the formal health sector has the potential to reduce service cost and improve availability of quality SRH (and other) care at the community level.

## Background

Many low-income countries are facing shortages of qualified health workforce [1], and there is an increasing interest in community health workers’ role as a bridge between communities and formal health-care systems [2]. “Community health worker” (CHW) is an umbrella term for those who provide health services at the level of the community where they (often) originate from [3]; there is a wide range of terminologies across and within countries used synonymously for CHW [4]. In this article, CHWs are referred to as close-to-community (CTC) health service providers who carry out promotional, preventive and/or curative health services and who are the first point of contact at the community level for health-related services. A CTC health service provider can be based in the community or in a basic primary care facility.

Although the primary goal of introducing CHWs was to enhance the accessibility and the affordability of health-care services in rural and poor urban communities, mainly for primary health care [4], in later years, the focus has been expanded to also include education, counselling, provision of preventive care and treating a limited range of common diseases [5]. In many low- and middle-income countries, CHWs are an important part of the health system, as they have a positive impact on certain health outcomes and constitute a cost effective approach of delivering health services at the community level [5].

Bangladesh has a pluralistic health system [6], and CHWs are an obvious resource within both government and non-governmental organization (NGO) sectors [7]. CHWs have been seen for several decades as an alternative to the complete professionalization of the health workforce in Bangladesh [8]. Both governmental and NGO CHWs are utilized for community health programmes, including family planning services, sexual and reproductive health (SRH) services, preventive services (for example, immunization and vitamin A distribution.) and several curative services (for example, management of childhood pneumonia, neonatal sepsis and TB control). Though both the government and NGOs run community health programmes, NGOs have been instrumental in scaling up these programmes [8].

Aside from CHWs, there is a wide range of informal providers in Bangladesh who can also be referred to as CTC health service providers. A national survey conducted in 2007 estimated qualified health practitioners, including physicians, dentists and nurses, as having a density of only 7.7 per 10 000 population, community health workers (mostly non-governmental) as having a density of 9.6 per 10 000 population, and paraprofessionals as having a density of 1 per 10 000 population [9]. At the same time, informal CTC health service providers, such as traditional healers (practitioners of traditional herbal medicine), *totka* practitioners (practitioners combining traditional and modern medicine) and faith healers, had an estimated density of 64.2 per 10 000 population [9]. In addition, traditional birth attendants, unqualified allopathic practitioners (village doctors and drugstore salesmen) and homeopath practitioners had an estimated density of 33.2, 23.9 and 5.9 per 10 000 population, respectively. Altogether, the estimated density of informal CTC health service providers was 127.2 per 10 000 population in Bangladesh in 2007, 12 times higher than that of the formal CTC health service providers. According to Cockcroft et al. [10], 60% of treatment services in rural Bangladesh are provided by informal providers.

A quasi-experimental research programme [11] in Matlab, a rural area of Bangladesh, found that beliefs about causes of illness and its cultural explanation was one of the key factors shaping women’s choice of preferred care provider. Accessibility to providers, cultural familiarity with the health problem and payment flexibility all play a major role in decision-making regarding choice of health service providers for women’s SRH problems [12]. Lack of basic health facilities, disrespect by providers, lack of accessibility and availability of formal qualified providers and the general unresponsiveness of the health system drive men and women from poor communities to informal providers [13]. Therefore, informal health-care providers play a huge and often under-recognized role in the Bangladeshi health systems, and their presence and importance require urgent increased attention.

There is increasing evidence on the important role of CTC health service providers in Bangladesh and in other low-income countries [8] where there is a shortage of qualified health workers [1]. In practice, the pluralistic nature of the Bangladeshi health system results in blurred public and private sectors, where many government doctors also have private practices and, in some cases, have smaller chambers in drugstores. The quality of diagnosis and care varies, and poor women and men often go back and forth between different kinds of providers [12].

Bangladesh is experiencing rapid urbanization [14]. Historically, formal CTC providers in Bangladesh largely served poor communities in rural areas [15], although NGOs have been instrumental in shifting the focus of these programmes to also include urban slums [8], which have expanded rapidly in the past decade [16]. Urban slum settlements make up 37.4% of the population in Dhaka city, while in most cities outside of Dhaka, many slum settlements are being established [17]. Within slums, the private health sector is poorly regulated, and there is lack of accountability in the public sector [9]. Informal CTC health service providers are, very often, the most accessible health-care providers, for example, they remain the main sources of family planning services in urban slums. They often maintain an informal or personal link to the formal health sector through referrals, which is usually based on personal relationships in a locality [18]. Understanding the interactions, communications and partnerships between CTC health service providers and the community is critical in this pluralistic provider context, in order to increase the number and appropriateness of referrals between providers, including referrals from the informal to the formal sector [19]. Gaps remain in the evidence base regarding the context in which different CTC SRH service providers operate in both urban slums and poor rural communities in Bangladesh.

The aim of this study was to understand the context in which the diverse group of CTC SRH providers operates in Bangladesh. The specific objectives were to explore the types of CTC SRH service providers accessed by women in poor slum and rural communities in Bangladesh, to better understand the factors influencing women’s choice of CTC providers for their SRH needs and to analyse the inter-relationships between formal and informal CTC SRH service providers and between these and the different communities they work in. Building this evidence base is important in this context, as CTC health service providers continue to expand and are extremely diverse in urban slums and rural communities in Bangladesh. This has implications for policy and future programme interventions related to the SRH needs of poor women.

## Methods

This qualitative study is part of a larger study conducted in Bangladesh by the James P Grant School of Public Health as part of the REACHOUT consortium (www.reachoutconsortium.org). The larger project is focused on improving the performance of CTC health service providers and the equity, effectiveness and efficiency of CTC health services available to poor women living in urban slums and rural communities. Four field sites – three urban slums and one rural area – were selected from two districts: Dhaka (the capital with the largest slum settlements in Bangladesh [17]) and Sylhet (a conservative, expanding urban slum with low-performing SRH indicators [20]). A health service provider’s mapping (conducted as part of our larger project in 2013, unpublished) in the selected sites found that only 1.6% of all (formal and informal) health service providers were qualified practitioners of allopathic medicine, while more than 68% were informal CTC providers, including unqualified allopathic practitioners (24.1%), traditional birth attendants (18.6%), traditional healers including herbalists and faith healers (21.9%), and informal homeopaths (3.8%). The rural site had the highest proportion (77%) of informal providers. Formal CTC providers constituted 23% of the health service providers; of these, 20.8% were NGO CHWs and 2.2% were government CHWs.

This paper presents data generated through in-depth interviews with menstrual regulation (that is, manual vacuum aspiration to safely establish non-pregnancy up to 8–10 weeks after a missed menstruation period [21]) clients, focus group discussions with married men and women in communities and semi-structured interviews with formal and informal CTC SRH service providers.

Twenty-four in-depth interviews were conducted with women who sought menstrual regulation any time over the previous year in order to understand women’s personal experiences and provider choice. For these in-depth interviews, we purposively selected participants from each of the following three categories: women who had never given birth (4), women who had had one or two children (10) and women who had had three or more children (10). All participants were married and aged between 17 and 42 years. An equal number of participants were selected from each of the following educational categories: no formal schooling and 1 to 5 years, 6 to 10 years and more than 10 years of formal schooling. These selection criteria enabled us to better understand the diversity of respondents’ experiences of menstrual regulation and other SRH services. Participants were interviewed at the respective clinics after they had received a follow-up service.

In addition to the in-depth interviews, we conducted 24 semi-structured interviews with formal (8) and informal (16) CTC SRH service providers. Depending on participants’ preference, these interviews were conducted either at their residence or place of work. Through these interviews, we learnt about formal and informal CTC SRH service providers’ perspectives on the services they provide. All providers were purposively selected based on their popularity from a list previously identified through a CTC health service provider mapping in 2013 (cited previously, unpublished) in the selected study sites. We also conducted 12 focus group discussions with married men (4) and women (8) in the community to explore SRH-related health-seeking behaviour. Each focus group discussion had 8 to 10 participants, who were selected from communities. Separate focus group discussions were organized for married men and women. In selecting participants for focus group discussions, special care was given to maintaining a relatively homogenous group to address potential adverse influences on group dynamics.

Data collection was completed between July and September 2013 in two slums in Dhaka city and one urban slum and one rural area in the Sylhet district. A 13-member research team was trained on qualitative data collection, data management using Atlas.ti and qualitative data analysis by experienced qualitative researchers. Transcripts were first written in Bangla (on the same day as the interview) and then translated into English. The translation process from Bangla to English was overseen by senior researchers to ensure quality. Data was coded using Atlas.ti following a coding frame drafted initially based on main study themes. Data triangulation was undertaken through comparing findings derived from different respondent groups, geographical locations and data collection tools, to ensure validity and reliability of the data.

Ethical approval of this study was obtained from the Royal Tropical Institute, the Netherlands (one of the consortium partners), and the James P Grant School of Public Health, Bangladesh, Research Ethical Committee. Informed consent was obtained from the informants before proceeding with data collection. Written consent was obtained from those who were literate, while illiterate participants gave their consent verbally. Confidentiality and anonymity were maintained throughout the research. Potential risks and benefits in participating in the study were discussed with the study participants, and it was explained that informants had every right to stop the interview at any point or to skip any questions they did not want to answer.

## Results

Our study findings showed that poor women visit a diverse range of CTC SRH service providers. We found that long-term relationships with informal CTC SRH service providers, trust and common understanding of illness influenced women’s SRH-related health-seeking behaviour. Treatment expenses and cooperative attitudes between formal and informal CTC SRH service providers also played a major role here.

### Range of and preferred CTC providers for SRH services

Data from focus group discussions revealed that women seek health services from a range of providers including formal (government, NGO and private sector) and informal (drugstore salesmen, traditional birth attendant and traditional healers) providers. This was confirmed by data from semi-structured interviews with formal and informal CTC SRH service providers and in-depth interviews with women who sought pregnancy termination services. Informal CTC health service providers were seen as the preferred providers for addressing women’s SRH-related problems at the community level. Availability of informal CTC SRH service providers, long-term relationships (which enabled the development of trusting relationships between women and informal providers) and cost implications of the service emerged as the key factors shaping women’s preference for informal CTC SRH service providers.

Analysis of focus group discussions with married men and women in urban slums and semi-structured interviews with CTC SRH service providers in Dhaka and Sylhet revealed that most respondents perceived nearby drugstores as the preferred provider for many women for general SRH services, which ranged from abdominal tenderness during menstruation to white discharge. Other preferred informal CTC SRH service providers included traditional healers and traditional birth attendants who provided both support and medicines. For example, a married woman, aged 22 years, working in a government tea estate explained:

“…during my period there is pain in my abdomen. When I had pain in my abdomen, then every month I bought painkillers from the pharmacy. When the pain subsides, then it’s no longer remembered. Next month I again take painkillers.” (Focus group discussion)

Many study participants reported that they visit formal health services when they are located close to their house and are easily accessible and familiar. For example, a married woman from the urban slum of Kallyanpur said that she goes to a drugstore first, and if the drugstore salesmen cannot help her, she then goes to a government hospital:

“We go to the pharmacies without consulting a doctor. We talk about our problems to the drugstore salesmen, and they give us medicines. If their medicines work, then we are saved. We feel that Allah has forgiven us. If we are not cured then we go to hospital X [a tertiary Government hospital].” (Focus group discussion)

As mentioned previously, informal CTC health service providers were preferred for SRH-related health services. However, women also visited hospitals if there was any complication. As mentioned by a 23-year-old married woman of Ghashitola, Sylhet:

“A dai [traditional birth attendant] came and attended the delivery of my baby. You have to give her a new saree [traditional Bangladeshi dress for women] and 200 taka. If somebody has a critical condition she has to go to a hospital.” (In-depth interview)

It is clear from our study that familiarity and ease of access and communication is an important factor in shaping health-seeking behaviour. Women prefer to speak about their ailments to local community providers, who are seen to speak the same cultural language of illness.

In the rural location of Lakkatura in Sylhet, focus group discussions with men and women revealed that some of the participants visited the government clinic inside their village, although lack of sufficient medicine and unavailability of a full-time doctor discouraged further visits. Thus, they preferred to visit available alternative health service providers, like drugstore salesmen and traditional healers, in Lakkatura for their health problems, including for SRH. According to a housewife, aged 31 years, in Sylhet:

“If a woman conceives, we take a Tabij [amulet] to protect the child. Then we would not face any problem. I brought a Tabij from my home, Mymansingh [another district]. My mother brought it for me. I wore it. I did not face any problem. The delivery was fine.” (Focus group discussion)

The perception of pregnancy care is shaped by cultural knowledge of what constitutes safe and unsafe behaviour. The use of local healers reinforces the belief that an amulet provides protection for the unborn child against supernatural spirits. This kind of cultural affinity with illness and health is also a key factor as to why local informal providers are preferred and trusted for particular ailments, such as pregnancy and miscarriage. We argue that local understanding of illness shapes and influences women’s health-seeking behaviour. Women felt comfortable when service providers spoke the same language and worked within similar cultural framings. Trust and ease of communication and access meant that informal providers, such as drugstore salesmen working in pharmacies, traditional healers and traditional birth attendants, were the most popular health service providers for SRH concerns and were the first point of contact for the community in both urban slums and rural settings.

In focus group discussions at the community level, women reported that they, and many others, visited Marie Stopes^1^ and the Reproductive Health Services Training and Education Programme (RHSTEP)^2^ for general pregnancy-related SRH services and for pregnancy termination services. The BRAC^3^ delivery centre, which provides services on family planning and mother and child care, was reported as another popular facility for formal CTC health service provision. Although some community members in urban slums mentioned that they visited government hospitals, they were not pleased with the services provided. In the focus group discussions, many of the men and women stated that women preferred not to go to government hospitals as a first choice since the quality of health services was considered to be poor. They mentioned complaints about the number of beds available for clients, long waiting hours, bribery and corruption and the influence of brokers at government hospitals. Brokers act as “middlemen” in some government hospitals. They roam around the hospital territory and request money from clients expecting to get admitted in the hospital or to get a doctor’s appointment. Sometimes, they also influence clients to go to private clinics for treatment as they get commission from the clinics. Within the focus group discussion in the urban slum of Keraniganj, participants discussed the role of brokers:

“You cannot get the doctor directly at hospital X [a government hospital] and it needs a broker. They take you to a doctor, and then the doctor will treat you. You do not need to look for them. They are available at the hospital gate.” (Married man, aged 58 years, Keraniganj, Dhaka, focus group discussion)

Many community members preferred to seek health care from informal CTC health service providers because of the multiple challenges they faced with formal providers and the easy access and availability of informal CTC health service providers in their locations.

### Influence of expenses on choosing CTC health service providers for SRH needs

Our study findings revealed that expenses for the SRH treatment had an influence on clients’ selection of CTC health service providers. As mentioned above, the presence of brokers in government hospitals, in both Dhaka and Sylhet, discouraged women from seeking SRH services from these facilities. The expenses for treatment in government hospitals also reduced community usage of formal government providers. For example, a woman in Dhaka explained:

“The government hospital charges 200/300 taka, but does not give good medicine. However, they take less money from the poor and from the rich people they take more. They do not give good medicine, so that patients [clients] have to visit them repeatedly.” (Married woman, aged 15 years, Keraniganj, Dhaka, focus group discussion)

Some informants suggested that government should provide subsidized menstrual regulation services for poor women. They argued that menstrual regulation services are expensive, and poor women struggle to pay high fees for this service. An in-depth interview informant explained:

“…It is better if this service is run by the government. … as we are poor people. We don’t have the ability to receive treatment from private clinics paying high fees. If the government take any initiative, taking in mind the issues of these poor people, then it would be good for everyone. Sister, you write this point down (with smiling face).” (Married woman, aged 35 years, Lakkatura, Sylhet)

Within the study area, treatment expense was a key factor shaping the preference for informal and NGO CTC health service providers over government providers. In five focus group discussions (two in Dhaka and three in Sylhet) and in one in-depth interview, respondents shared that many female community members went to NGOs for their SRH-related health problems since they either provided free services or used discounted rates. However, other participants mentioned that although NGOs were expected to offer less expensive health services, this often was not the case. For example, a married woman in Kallyanpur explained:

“I do not feel good about the health workers of clinic X [an NGO clinic]. Whenever I get the chance to face them… they even charge a huge amount of money.” (Married woman, age not mentioned, Kallyanpur, Dhaka, focus group discussion)

The same woman reported that NGOs take money, but the clients do not receive quality services in return:

“They take money but do not give [family planning] injections according to the scheduled dates. They say ‘not today, come tomorrow; not tomorrow, come day after tomorrow’ and things like this.” (Focus group discussion)

In 7 out of 12 focus group discussions, participants mentioned that women first visit drugstore salesmen for SRH problems for low-cost or minimal-cost services. These can include methods for family planning, treatment for pregnancy pain and request for pills for pregnancy termination. Traditional healers were also preferred since they were the cheapest option for clients; in many cases, traditional healers did not charge money. A 39-year-old married woman in Lakkatura in Sylhet explained this as follows:

“Herbal medicine is what we take. We hope that we will get cured using a smaller amount of money. If we spend too much money, we then have to starve to death. Then nobody will take care of us. And then again if we start owing a lot of money, it creates a lot of mental pressure on us.” (Focus group discussion)

In another case, a 30-year-old married woman from the urban slum of Ghashitola in Sylhet referred to the case of her neighbour who went to a government hospital in Sylhet for SRH-related treatment but, due to the high expenses, went back to a traditional healer:

“She could not bear the expenses of hospital X [a tertiary government hospital] anymore, so she went back home. She was under a lot of debt. Later, she visited a kabiraj [traditional healer], and the kabiraj said she was cursed. She had to spend a lot to break the curse.” (Focus group discussion)

### Trust and relationship influence client access for CTC SRH service providers

Payment method reflected the clients’ relationship with their health service providers. As mentioned in the above section, formal health service providers were always paid in cash. However, informal health service providers sometimes received gifts as a token of gratitude for their services. Informal CTC health service providers welcomed non-monetary payments and payments on credit. Some informal CTC health service providers mentioned that they valued the respect and trust they got from the community people, claiming that it was worth more than money to them. A drugstore salesman from the urban slum in Sylhet explained:

“Patients [clients] send me Panjabi [traditional Bangladeshi dress for men] during Eid as a gift. Some patients [clients] take me to their home to have a cup of tea.” (Drugstore salesman, aged 55 years, Ghashitola, Sylhet, semi-structured interview)

Another informal CTC service provider also expressed the same. According to her:

“Few days ago I attended a delivery at Amtala and it was a baby girl. They gave me a saree [traditional Bangladeshi dress for women] and fed me milk with rice. Listen; when you die you cannot take money with you so what’s the benefit to take money forcefully?” (*Dai*, aged 65 years, in practice for 50 years, Kallyanpur, Dhaka, semi-structured interview)

These illustrate that monetary matters are not always important to informal CTC health service providers to provide their services; rather, they emphasized the relationship they have with their clients. This good relationship was expressed through the gifts they received from their clients.

In interviews with CTC health service providers, informants emphasized the importance of community respect and trust in terms of service delivery. It was revealed that having or perceiving community trust motivated CTC providers to perform their duties better and allowed them better access in the community. Informal CTC health service providers developed a range of strategies to gain this trust within communities. According to one of the informal CTC health service providers in Dhaka:

“I maintain good relationships with those who come to me. They share with me their mental sufferings. …It is also seen that they have family problems. There are so many things which they can’t share with anyone. So they come to me and say all those things.” (Drugstore salesmen, aged 29 years, in practice for 18 months, Kallyanpur, Dhaka, semi-structured interview)

An unqualified practitioner of allopathic medicine from the rural site of Sylhet, who was 50 years old and was in practice for 25 years, mentioned:

“the relation is very good. They trust me. They love me. Everyone from the garden comes to me. Everyone of Lakkatoorah comes here. If no one loved me, believed me or respected me, would they come to me? Everyone comes to me because they love me, trust me, and respect me. There’s no medicine in the house. Capital is needed for keeping medicine in the house. Capital is needed. There are only things for primary treatment in the house.” (Semi-structured interview)

Informal CTC health service providers perceived that they have a trusting set of relationships and reputation in the community, which in turn brings a strong motivation to work and to provide good services. A 50-year-old traditional birth attendant, in practice for 30 years, explained:

“… I go out at 2.00 am too. No one says anything. Even the Mafias [referring to the leaders of local thugs] don’t say anything to me. Their children’s delivery also happens by my hand. They know that they need me. If I go somewhere late at night, they understand that I have a delivery to attend. That day I went to Kolapara at 2.00am. On the way I met a Mastan [a community thug or influential people sometimes backed by the political parties]. He asked me, ‘Aunty, where are you going?’ I told him, ‘Kolapara, a patient’s [client’s] house.’ He told me, ‘You can go, Aunty. There’s no problem. If there’s any problem, just tell them my name.’ Then I said, ‘You guys are the Mastans. If you don’t do any harm to me, who else would do it?’ I talked like this. They respect me. That’s why he didn’t say anything.” (Ghashitola, Sylhet, semi-structured interview)

For the formal CTC health service providers, the scenario for gaining the trust from the community was different. They maintained a good relationship with community leaders and with influential community representatives, including local government and teachers. This strategy helped them to gain access to the community. In this regard, a formal CTC SRH service provider of an urban slum of Sylhet, aged 45 years and in practice for 20 years, explained:

“At first I need to build up a relationship with the patient [client] before trying to bring her [to my clinic]. I cannot convince her even if I tell her a thousand times that BRAC provides good service, if I cannot build up a good relationship with her with good behaviour. I need to build up trust first. So that she believes whatever I tell her. To build up relationship I need to talk about a lot of different topics at first. If she is cooking at the time I go there, I start by asking her things like what she is cooking, how the children are doing, how many children she has etc. And after talking about these things for a while I move to health related topics at the last moment.” (Semi-structured interview)

Considering the community context, four respondents (both formal and informal CTC SRH service providers – namely, a family welfare assistant, a traditional healer and a centre manager and a programme officer from Marie Stopes) from the urban slums of Kallyanpur and Keraniganj in Dhaka and Ghashitola in Sylhet explained that having a strong relationship is very important to carry out the services for the community. An experienced traditional healer, aged 50 years, in practice for 12 years, from the urban slum of Ghashitola, Sylhet, stated that his relationship with clients acts as a facilitator to receive more clients from the community:

“I have good relationships with my patients [clients]. They would not come to me if the relationship was not good. … Because of this good relationship one patient [client] brings in another five patients [clients].” (Semi-structured interview)

Community trust and familiarity are developed over a period of time by maintaining a good relationship with clients and through the services offered within a community setting. Gaining trust is particularly important for providing awareness services on SRH-related issues to adolescents and newly married couples. For example, a family welfare assistant mentioned in her interview that it was very difficult for her to promote SRH-related information to newlywed couples who were not open or receptive to sharing or learning about SRH issues.

### Interaction between formal and informal CTC SRH service providers

Our data revealed that there was a multifaceted interaction among the formal and informal CTC SRH service providers. Some formal CTC providers mentioned that a good relationship was maintained with informal providers. This relationship, which developed informally, helped them to learn from each other and also to get support from other CTC SRH service providers if needed. Thus, a formal CTC SRH service provider from Ghashitola, Sylhet, said:

“I have a good relationship with them [traditional birth attendants]. I get help and support from them when I am in trouble [in providing health services].” (Aged 25 years and in practice for 2 1/2 years, semi-structured interview)

Links between formal and informal health-care service providers also secured health services for their clients and, often, timely care. For example, an informal CTC SRH service provider mentioned that she had a good relationship with formal providers; they supported each other and thus ensured better services to their clients. According to her:

“Sometimes during delivery, if the cervical opening was not even three fingers open; the woman would be given injections. But the doctors used to avoid injections. They were not willing to take risk. … They also discourage me. They say, “Khala [aunty], don’t do such risky things [giving injection]. Take the patient [client] to the hospital. Check if the cervical opening is clear, if the baby is in good position.” (Traditional birth attendant, aged 65 years, in practice for 50 years, Kallyanpur, Dhaka, semi-structured interview)

In this scenario, it can be seen that both formal and informal CTC health service providers cared for their clients and provided services for women for SRH-related health problems. Cooperative attitudes between formal and informal health service providers can reduce harmful practices when treating women’s SRH health problems and ensure better services.

## Discussion

Deploying different qualitative methods in our study area confirmed that poor women living in urban slums and rural areas visit a diverse range of CTC health service providers for their SRH-related health problems, with the informal sector being preferred over the formal sector. Women do not choose providers because they are “formal” or “informal”; rather, their preference is influenced by availability and accessibility of services, treatment expenses, easy communication, cultural familiarity, trust and respect, as well as the behaviour of different CTC health service providers.

Within the Dhaka slums, women tend to visit informal providers initially, such as drugstore salesmen, traditional healers or traditional birth attendants, before (if perceived necessary) going to hospitals. Drugstore salesmen, traditional birth attendants and traditional healers were also very popular among poor women in Sylhet for SRH services because of the similar reasons of ease of access, familiarity and low treatment expenses. A previous study on the reproductive and sexual health-care market in two rural and one urban slum in Bangladesh, in which participants were also married women, found similar trends in terms of choice of providers. It is noteworthy that both of these studies have one common rural study area [12].

Most of the participants first visited a drugstore to obtain SRH-related services, confirming findings from Ahmed et al. [22], who argue that drugstore salesmen are emerging as one the most popular choices for health services in Bangladesh. Availability and low treatment expenses are key factors that shape female clients’ preference for providers in our study context. Our findings also reveal that many participants accessing formal health service providers, especially at government facilities, often needed brokers who act as a link between formal providers and clients and charge clients for their services. Costs associated with formal health services are comparatively higher, and the unavailability of drugs at the facility (which is perceived as offering poor services) is the main reasons for not choosing formal care as a first point of contact. The method of payment to the health service providers shaped relationships between health service providers and clients. Clients need to pay the formal health service (CTC) providers in cash, while for the informal CTC health service providers, remuneration is not always monetary and can also include in-kind payments and payments on credit. Our findings are supported by a study conducted in 2008–2009 that found low treatment expenses and flexibility in payment methods (for example, credit and alternate payment methods like food) as factors that, among others, attract poor women to informal providers [12].

In this study, trust between clients and providers was also found to be an important factor for both formal and informal providers, in the context of their relationship with clients. Provider–client relationships and communication are important to meet the non-medical expectations of clients [23]. In all four research sites, most informal providers were able to gain the trust of the community and build positive relationships. In line with Helman [23], we argue that since informal providers are mostly from the community, they are acknowledged within the local context and that this provides cultural and social familiarity and a “comfort zone” for clients to interact with providers regarding their SRH problems. A study by Rashid et al. [12] confirms that ease of access and communication are important factors in shaping health-seeking behaviour. They argue that lack of hierarchy and absence of class differences made women feel at ease in accessing informal CTC providers for their SRH-related health problems. Another qualitative study [24] in Bangladesh has shown that cultural affinity with illness and health is also a key factor as to why local informal providers are preferred and trusted for particular ailments (such as pregnancy complications and miscarriage). On the other hand, formal health service providers face challenges in getting access to the community and gaining and sustaining trust at the community level. This is because they are not from the community, hence are not considered as their “family”. These formal providers have to work hard to build rapport with and gain the trust of the community in order to provide SRH and other necessary services, as most people with SRH problems visit informal providers more frequently [22, 25]. An anthropological study on childless women in Bangladesh reveals that women prefer informal service providers for their SRH-related health services. This preference is facilitated by sex of the providers, their availability and expenses for the services. Moreover, informal health service providers and women share the same understanding of illness that eases women to express their problem [26].

Formal CTC SRH service providers try to maintain a good relationship with community leaders and influential community members (elected local government representatives and teachers) as it allows them to gain better access to the community. Non-financial support, respect, social prestige and positive community feedback were particularly welcomed by informal CTC SRH service providers, confirming findings of a study by Alam et al. [27] which assessed the factors relating to the retention of female volunteer community health workers in the Dhaka urban slums.

In terms of interaction between formal and informal providers, some informal communication at a professional level was reported by both groups. Most formal providers maintain a liaison with informal providers in order to obtain and retain more clients, whereas most informal providers prefer to work independently. Both groups recognize the benefits of interacting with each other and the positive effect on SRH-related services for clients. A study in a rural area of Peru [19] argues that improved interaction, comprehension and understanding between formal and informal providers positively influences the referral process and supports SRH-related service provision. Improving this communication and the links between different types of formal providers and between formal and informal providers has the potential to facilitate appropriate and timely referral and better meet the poor women’s SRH needs

### Limitations of the study

In-depth interview participants were interviewed at the respective clinics after they had received a pregnancy termination follow-up service. They were not recruited from the community or interviewed in the community because during field testing we experienced difficulties in locating women who sought pregnancy termination service there. We also observed that participants were reluctant to discuss pregnancy termination and other SRH issues at home, because of the fear that discussion maybe overheard by other family members or neighbours. This challenge was largely avoided in a clinic setting. The majority of our in-depth interview respondents had visited informal providers to discuss or receive pregnancy termination services before they visited a formal health facility. However, because of our selection strategy, we missed women who received pregnancy termination services only from informal providers and hence their experiences are not included. The issue of trust and relationship between different CTC providers and community was addressed from providers’ perspectives only as we missed including the voices of clients here. Further studies should explore service users’ voice in this regard.

## Conclusion

Informal CTC providers dominate health services in poor urban and rural communities and are often preferred by women for their SRH needs over formal CTC providers. They are usually the first health-care point of contact, even for those who later on access SRH services from formal providers. However, there is no effective link between formal and informal providers in terms of referral, coordination and communication regarding SRH services. Cooperation and good understanding among formal and informal health service providers could lead to the provision of better SRH services. Training informal CTC health service providers and developing strategies to enable better links and coordination between this culturally embedded and accessible cadre and formal (NGO and government) health sectors have the potential to reduce service cost and make quality SRH (and other) care more available and accessible at the community level.

## Abbreviations

CHW: Community health worker
CTC: Close-to-community
NGO: Non-governmental organization
RHSTEP: Reproductive Health Services Training and Education Programme
SRH: Sexual and reproductive health

## Footnotes

1 Marie Stopes is a NGO which provides SRH services especially for the poor and vulnerable (http://www.mariestopes-bd.org/).

2 RHSTEP is an NGO providing sexual, reproductive and general health services to women, men, adolescents and children (http://www.rhstep.org/).

3 BRAC is a development organization dedicated to alleviate poverty by empowering the poor and helping them to bring about positive changes in their lives by creating opportunities for the poor (http://www.brac.net/).
